# FROM THE EDITOR 

**DOI:** 10.18699/VJGB-23-84

**Published:** 2023-12

**Authors:** N.А. Kolchanov, Yu.G. Matushkin

**Affiliations:** Institute of Cytology and Genetics of the Siberian Branch of the Russian Academy of Sciences, Novosibirsk, Russia; Institute of Cytology and Genetics of the Siberian Branch of the Russian Academy of Sciences, Novosibirsk, Russia

Dear colleagues, dear readers! We present to your attention a new issue of the Vavilov Journal of Ge netics and Breeding dedicated to bioinfor matics and systems computational biology. These areas of scientific research are now rapidly transforming as natural sciences enter the era of big data. Intense develop ment of the omics technologies (genomics, transcriptomics, proteomics, metabolomics) and other high throughput technologies for studying molecular and genetic foundations of living systems’ functioning has led to an information explosion in genetics, which is the main source of big data in world science, ahead of the other sciences and technologies in terms of the rate and volume of experi mental data accumulation

An important result of the analysis, interpretation and understanding of big genetic data is the formation of a new paradigm, wherein the main objects of genetics are not separate genes, but gene networks – groups of genes functioning in coordination, interacting with each other through their products, such as RNA, proteins, metabolites and other substances. It is gene networks that ensure the formation of all phenotypic (molecular, biochemical, cellular, physiological, morphological, behavioral, psychological, etc.) features of organisms based on the information coded in their genomes (Kolchanov et al., 2000, 2013; Ananko et al., 2002).

Reconstruction of gene networks is a very complex task requiring a search, extraction and integration of informa tion scattered across tens of millions of scientific articles, thousands of factographic databases and millions of patents containing biological, medical, pharmacological, chemical, and other knowledge. To solve this task, it was necessary to develop computer software systems for automatic extraction of genetic data from the aforementioned sources using a combi nation of traditional textual analysis and methods of machine learning (Ivanisenko V.A. et al., 2019; Ivanisenko T.V. et al., 2022). To this day, more than 70,000 gene networks and their main components (signaling pathways, protein protein, DNA protein, RNA protein interaction networks, metabolic pathways) have been reconstructed and presented in databases (Pico et al., 2008; Caspi et al., 2020; Kanehisa et al., 2023).

Accumulation of big data has resulted in the understand ing of the great complexity of gene networks regulation on the base levels of their organization: each elementary fundamental biochemical or molecular biological process in a gene network is usually controlled by dozens, sometimes hundreds of elementary regulatory processes, whether it con cerns protein enzyme activity, gene transcription regulation or “regulation of complex metabolic pathways” (Kolchanov et al., 2008). The abovementioned makes it incredibly difficult to reconstruct molecular mechanisms of the influence of ge nomic variability on phenotypic characteristics of organisms and clinical disease symptoms due to the fact that, among other things, regulatory processes are often characterized by a high degree of nonlinearity (Costanzo et al., 2019; Trifonova et al., 2021; Pratap et al., 2022) and dynamic instability in relation to changes in the initial data and constant physico chemical and molecular biological processes underlying the functioning of gene networks and regulatory systems (Khlebodarova et al., 2018).

Processing, analysis and interpretation of big genetic data streams requires the development of modern artificial intel ligence methods focused on living systems. A key event that has initiated the rapid development of artificial intelligence methods in recent years is the creation of a new architecture of neural networks called transformers, which are geared towards the processing of symbol sequences, including texts in natural languages (Vaswani et al., 2017). The main feature of transformers is that the order of input sequences during processing is irrelevant. This provides ample opportunities for paralellizing, allowing for the deep learning of models on terabytes of data in a much shorter time than was previously possible using classic neural network architecture.

Let us note a few remarkable achievements of this approach. The creation of high quality systems of machine translation from one natural language into another is of key importance (Jiao et al., 2023; Wang et al., 2023). The meaning of this development for science, technology, culture, art and human communication cannot be overestimated.

Based on transformer models, a huge breakthrough was made in solving one of the central tasks of molecular bio logy, which had been puzzling physicists, chemists and biologists for 60 years – predicting the spatial structure of globular proteins by their amino acid sequences. To solve this task, the AlphaFold (Thornton et al., 2021) and Rosetta (https://www.rosettacommons.org/) neural networks, predic ting the 3D coordinates of heavy protein atoms with precision close to experimental, were developed. The network learning was based on hundreds of thousands of proteins with a known spatial structure and tens of millions amino acid sequences.

The methods of machine learning using transformer ap proaches created an opportunity for modeling the dynamics of complex molecular biological structures containing a large (up to 109) number of atoms (Pandey et al., 2022). These results are significant not only for fundamental science but also for a wide range of areas with a big potential for practical application, such as biotechnologies, genetics, medicine, pharmacology, creation of new materials, and many others

Since 2017, when first publications on transformer tech nologies appeared, there has been an exponential growth of the number of publications using artificial intelligence methods (Eraslan et al., 2019; Boudry et al., 2022). Another machine learning approach that has been widely used and developed in the last years is graph neural networks (GNN), which provide entirely new opportunities for analysis of complex network structures based on the vector representation of graph vertices taking into account their local environment (Hamilton et al., 2017). The use of GNN is efficient for description, analysis and modeling of a wide range of network systems, be they natural, anthropogenic or technical: gene networks, intermolecular interaction networks, knowledge networks, social networks, etc. (Ektefaie et al., 2023).

In conclusion, it should be noted that there is a crucial limi tation to a wide application of artificial intelligence methods in the areas of human activity that have a practical significance: its opaque decision making process. In a number of works (Ma et al., 2018), a strategic way to overcome this restriction has been shown: it is necessary to develop hybrid information systems of a new generation, integrating classic methods of bioinformatics and systems computational biology and new artificial intelligence technologies based on the ontological description of the subject areas of research. In our opinion, only such an approach can ensure both the speed and quality of big genetic data processing with the use of artificial intel ligence methods, and the transparency of the results obtained.

## Conflict of interest

The authors declare no conflict of interest.
